# Synergistic growth inhibition by acyclic retinoid and phosphatidylinositol 3-kinase inhibitor in human hepatoma cells

**DOI:** 10.1186/1471-2407-13-465

**Published:** 2013-10-08

**Authors:** Atsushi Baba, Masahito Shimizu, Tomohiko Ohno, Yohei Shirakami, Masaya Kubota, Takahiro Kochi, Daishi Terakura, Hisashi Tsurumi, Hisataka Moriwaki

**Affiliations:** 1 Department of Gastroenterology, Gifu University Graduate School of Medicine, Graduate School of Medicine, 1-1 Yanagido, Gifu 501-1194, Japan

**Keywords:** Acyclic retinoid, LY294002, Hepatocellular carcinoma, RXRα, Synergism

## Abstract

**Background:**

A malfunction of RXRα due to phosphorylation is associated with liver carcinogenesis, and acyclic retinoid (ACR), which targets RXRα, can prevent the development of hepatocellular carcinoma (HCC). Activation of PI3K/Akt signaling plays a critical role in the proliferation and survival of HCC cells. The present study examined the possible combined effects of ACR and LY294002, a PI3K inhibitor, on the growth of human HCC cells.

**Methods:**

This study examined the effects of the combination of ACR plus LY294002 on the growth of HLF human HCC cells.

**Results:**

ACR and LY294002 preferentially inhibited the growth of HLF cells in comparison with Hc normal hepatocytes. The combination of 1 μM ACR and 5 μM LY294002, in which the concentrations used are less than the IC_50_ values of these agents, synergistically inhibited the growth of HLF, Hep3B, and Huh7 human HCC cells. These agents when administered in combination acted cooperatively to induce apoptosis in HLF cells. The phosphorylation of RXRα, Akt, and ERK proteins in HLF cells were markedly inhibited by treatment with ACR plus LY294002. Moreover, this combination also increased RXRE promoter activity and the cellular levels of RARβ and p21^CIP1^, while decreasing the levels of cyclin D1.

**Conclusion:**

ACR and LY294002 cooperatively increase the expression of RARβ, while inhibiting the phosphorylation of RXRα, and that these effects are associated with the induction of apoptosis and the inhibition of cell growth in human HCC cells. This combination might therefore be effective for the chemoprevention and chemotherapy of HCC.

## Background

Retinoids, vitamin A metabolites and analogs, are ligands of the nuclear receptor superfamily that exert fundamental effects on cellular activities, including growth, differentiation, and death (regulation of apoptosis). Retinoids exert their biological functions primarily by regulating gene expression through 2 distinct nuclear receptors, the retinoic acid receptors (RARs) and retinoid X receptors (RXRs), which are ligand-dependent transcription factors [1,2]. Among retinoid receptors, RXRs are regarded as master regulators of the nuclear receptor superfamily because they play an essential role in controlling normal cell proliferation and metabolism by acting as common heterodimerization partners for various types of nuclear receptors [1,2]. Therefore, altered expression and function of RXRs are strongly associated with the development of various disorders, including cancer, whereas targeting RXRs by retinoids might be an effective strategy for the prevention and treatment of human malignancies [3].

Hepatocellular carcinoma (HCC) is one of the most frequently occurring cancers worldwide. Recent studies have revealed that a malfunction of RXRα, one of the subtypes of RXR, due to aberrant phosphorylation by the Ras/mitogen-activated protein kinase (MAPK) signaling pathway is profoundly associated with liver carcinogenesis [4-9]. On the other hand, a prospective randomized study showed that administration of acyclic retinoid (ACR), a synthetic retinoid which targets RXRα, inhibited the development of a second primary HCC, and thus improved patient survival from this malignancy [10,11]. ACR inhibits the growth of HCC-derived cells via the induction of apoptosis by working as a ligand for retinoid receptors [12,13]. ACR also suppresses HCC cell growth and inhibits the development of liver tumors by inhibiting the activation and expression of several types of growth factors and their corresponding receptor tyrosine kinases (RTKs), which lead to the inhibition of the Ras/MAPK activation and RXRα phosphorylation [8,9,14-17]. These reports strongly suggest that ACR might be a promising agent for the prevention and treatment of HCC.

Phosphatidylinositol 3-kinase (PI3K) is activated by growth factor stimulation through RTKs and Ras activation, and plays a critical role in cell survival and proliferation in collaboration with its major downstream effector Akt, a serine-threonine kinase [18-20]. Increasing evidence has shown that aberrant activation of the PI3K/Akt pathway is implicated in the initiation and progression of several types of human malignancies, including HCC, indicating that targeting PI3K/Akt signaling might be an effective strategy for the treatment of cancers [18-22]. Several clinical trials have been conducted to investigate the safety and anti-cancer effects of therapeutic agents that inhibit the PI3K/Akt signaling cascade [18-20]. Combined treatment with a PI3K/Akt inhibitor and other agents, including MAPK inhibitors, might also be a promising regimen that exerts potent anti-cancer properties [23,24].

Combination therapy and prevention using ACR as a key drug is promising for HCC treatment because ACR can act synergistically with other agents in suppressing growth and inducing apoptosis in human HCC-derived cells [17,25-30]. The aim of the present study is to investigate whether the combination of ACR plus LY294002, a PI3K inhibitor, exerts synergistic growth inhibitory effects on human HCC cells, and to examine possible mechanisms for such synergy, predominantly focusing on the inhibitory effects on RXRα phosphorylation by a combination of these agents.

## Methods

### Materials

ACR (NIK-333) was supplied by Kowa Pharmaceutical (Tokyo, Japan). LY294002 was purchased from Wako (Osaka, Japan). Another PI3K inhibitor NVP-BKM120 (BKM120) was from Selleck Chemicals (Houston, TX, USA).

### Cell lines and cell culture conditions

HLF, Huh7, Hep3B, and HepG2 human HCC cell lines were obtained from the Japanese Cancer Research Resources Bank (Tokyo, Japan) and were maintained in Dulbecco’s Modified Eagle Medium (DMEM) supplemented with 10% FCS and 1% penicillin/streptomycin. The Hc human normal hepatocyte cell line was purchased from Cell Systems (Kirkland, WA, USA) and maintained in CS-S complete medium (Cell Systems). These cells were cultured in an incubator with humidified air containing 5% CO_2_ at 37°C.

### Cell proliferation assays

Three thousand HCC (HLF, Huh7, Hep3B, and HepG2) or Hc cells were seeded on 96-well plates in serum-free medium. Twenty-four hours later, the cells were treated with the indicated concentrations of ACR or LY294002 for 48 hours in DMEM supplemented with 1% FCS. Cell proliferation assays were performed using a MTS assay (Promega, Madison, WI, USA) according to the manufacturer’s instructions. The combination index (CI)-isobologram was used to determine whether the combined effects of ACR plus LY294002 were synergistic [25,27,30,31]. HLF cells were also treated with a combination of the indicated concentrations of ACR and BKM120 for 48 hours to examine whether this combination synergistically inhibited the growth of these cells.

### Apoptosis assays

Terminal deoxynucleotidyl transferase-mediated dUTP nick-end labeling (TUNEL) and caspase-3 activity assays were conducted to evaluate apoptosis. For the TUNEL assay, HLF cells (1 × 10^6^), which were treated with 1 μM ACR alone, 5 μM LY294002 alone, or a combination of these agents for 48 hours, were stained with TUNEL methods using an In Situ Cell Death Detection Kit, Fluorescein (Roche Diagnostics, Mannheim, Germany) [25]. The caspase-3 activity assay was performed using HLF cells that were treated with the same concentrations of the test drugs for 72 hours. The cell lysates were prepared and the caspase-3 activity assay was performed using an Apoalert Caspase Fluorescent Assay Kit (Clontech Laboratories, Mountain View, CA, USA) [30].

### Protein extraction and western blot analysis

Protein extracts were prepared from HLF cells treated with 1 μM ACR alone, 5 μM LY294002 alone, or a combination of these agents for 12 hours because this treatment time was appropriate for evaluating the expression levels of phosphorylated extracellular signal-regulated kinase (p-ERK), phosphorylated Akt (p-Akt), and phosphorylated RXRα (p-RXRα) proteins [25,29,30]. Equivalent amounts of extracted protein were examined by western blot analysis using specific antibodies [25]. The anti-RXRα and anti-RARβ antibodies were from Santa Cruz Biotechnology (Santa Cruz, CA, USA). The primary antibodies for ERK, p-ERK, Akt, p-Akt, and glyceraldehyde 3-phosphate dehydrogenase (GAPDH) were from Cell Signaling Technology (Beverly, MA, USA). The antibody for p-RXRα was kindly provided by Drs. S. Kojima and H. Tatsukawa (RIKEN Advanced Science Institute, Saitama, Japan).

### RNA extraction and quantitative RT-PCR analysis

Total RNA was isolated from HLF cells using an RNAqueous-4PCR kit (Ambion Applied Biosystems, Austin, TX, USA) and cDNA was amplified from 0.2 μg of total RNA using the SuperScript III Synthesis system (Invitrogen, Carlsbad, CA, USA) [32]. Quantitative real-time reverse transcription PCR (RT-PCR) analysis was performed using specific primers that amplify the RARβ, p21^CIP1^, cyclin D1, and β-actin genes. The specific primer sets used have been described elsewhere [25,30].

### RXRE reporter assays

HLF cells were transfected with RXR-response element (RXRE) reporter plasmids (100 ng/well in 96-well dish), which were kindly provided by the late Dr. K. Umesono (Kyoto University, Kyoto, Japan), along with p*RL*-CMV (*Renilla* luciferase, 10 ng/well in 96-well dish; Promega) as an internal standard to normalize transfection efficiency. Transfections were carried out using Lipofectamine LTX Reagent (Invitrogen). After exposure of cells to the transfection mixture for 24 hours, the cells were treated with 1 μM ACR alone, 5 μM LY294002 alone, or a combination of these agents for 24 hours. The cell lysates were then prepared, and the luciferase activity of each cell lysate was determined using a dual-luciferase reporter assay system (Promega) [25].

### Statistical analysis

The data are expressed in terms of means ± SD. The statistical significance of the differences in the mean values was assessed using one-way ANOVA, followed by Tukey-Kramer multiple comparison tests. Values of <0.05 were considered significant.

## Results

### ACR and LY294002 cause preferential inhibition of growth in HLF human HCC cells in comparison with Hc normal hepatocytes

**Figure 1:**
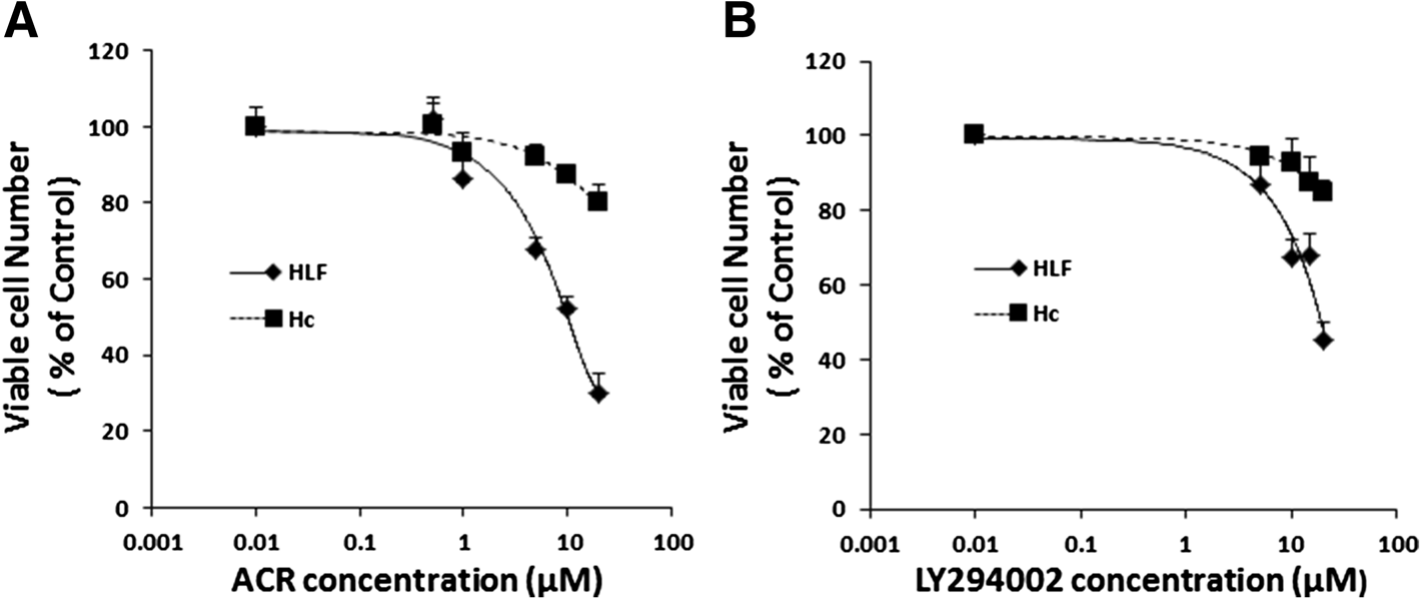
**Inhibition of cell growth by ACR and LY294002 in HLF human HCC cells and Hc normal hepatocytes.** HLF and Hc cells were treated with the indicated concentrations of ACR **(A)** or LY294002 **(B)** for 48 hours. Cell viability was determined by the MTS assay and expressed as a percentage of the control value. Error bars present the SD of triplicate assays.

In the initial study, the growth inhibitory effect of ACR and LY294002 on HLF human HCC cells and on Hc hepatocytes was examined. ACR (Figure 1A) and LY294002 (Figure 1B) inhibited the growth of HLF cells with IC_50_ values of approximately 6.8 μM and 15 μM, respectively. On the other hand, Hc cells were resistant to these agents because the IC_50_ values of ACR and LY294002 for the growth inhibition of Hc cells were each greater than 50 μM (Figure 1). These results suggest that ACR and LY294002 preferentially inhibit the growth of HCC cells compared with that of normal hepatocytes.

### ACR along with LY294002 causes synergistic inhibition of growth in HCC cells

**Figure 2:**
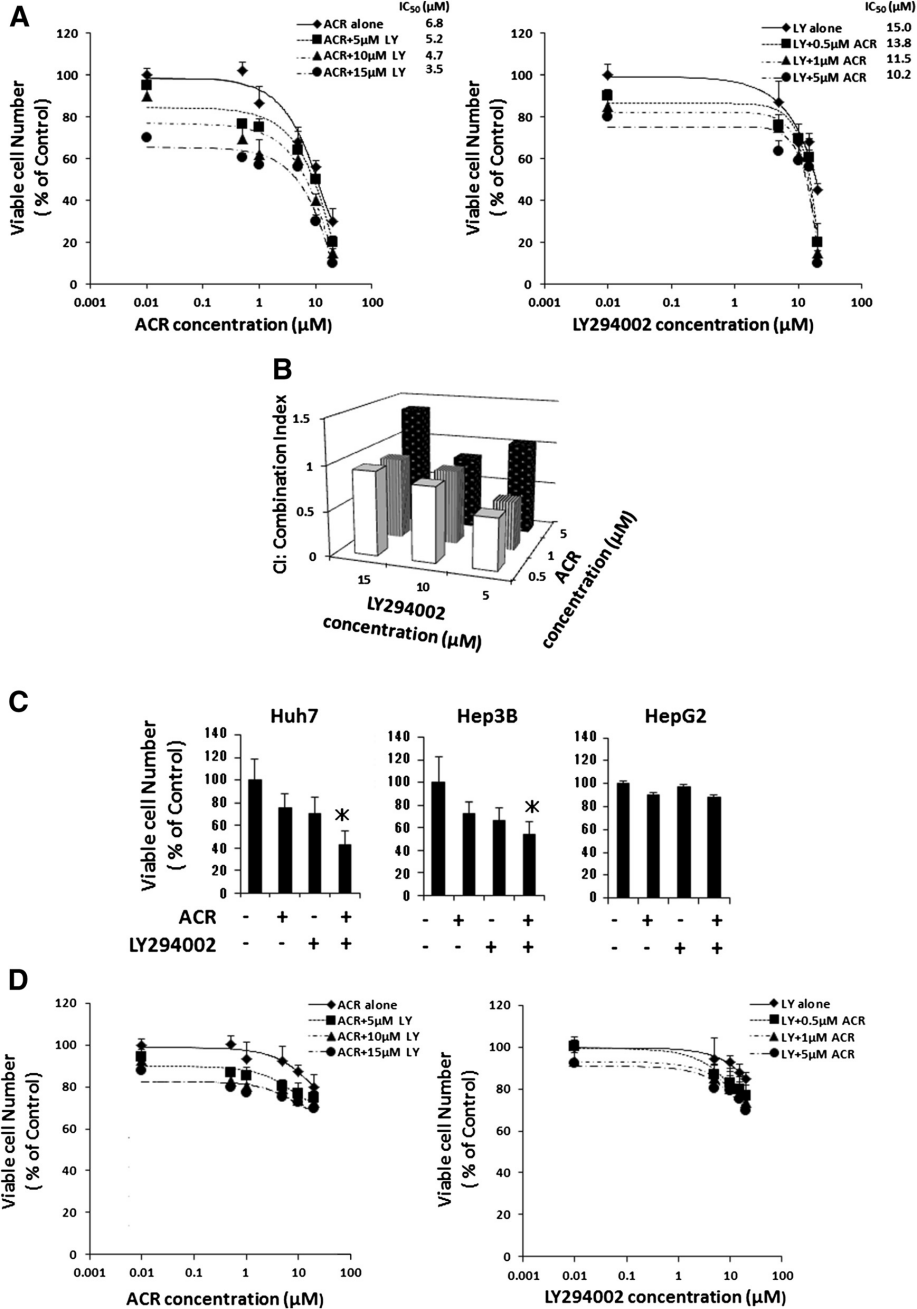
**Inhibition of cell growth by ACR alone, LY294002 alone, or various combinations of these agents in human HCC-derived cells and Hc normal hepatocytes. (A)** HLF human HCC cells were treated with the indicated concentrations of ACR alone, LY294002 alone, and various combinations of these agents for 48 hours. **(B)** The data obtained in **(A)** was used to calculate the combination index. **(C)** Huh7, Hep3B, and HepG2 human HCC cells were treated with vehicle, 1 μM ACR alone, 5 μM LY294002 alone, or a combination of 1 μM ACR and 5 μM LY294002 for 48 hours. **(D)** Hc human hepatocytes were treated with the indicated concentrations of ACR alone, LY294002 alone, and various combinations of these agents for 48 hours. **(A)**, **(C)**, and **(D)** Cell viability was determined by the MTS assay and expressed as a percentage of the control value. Error bars present the SD of triplicate assays. * *P* < 0.05.

Next, the effects of the combined treatment of ACR plus LY294002 on the growth of HCC-derived cells and Hc hepatocytes were examined. When HLF human HCC cells were treated with a range of concentrations of these agents, the CI indices for less than 1 μM ACR (0.5 or 1 μM) plus less than 10 μM LY294002 (5 or 10 μM) were 1+ (slight synergism), 2+ (moderate synergism), or 3+ (synergism). In particular, the combination of as little as 1 μM ACR (approx. IC_15_ value) and 5 μM LY294002 (approx. IC_25_ value) exerted synergistic growth inhibition because the CI-isobologram analysis yielded a CI index of 0.54 (3+), which indicates synergism [25,27,30,31], with this combination (Figure 2A,B, and Table 1). In other HCC cell lines, including Huh7, Hep3B, and HepG2 cell lines, similar findings were also obtained using Huh7 and Hep3B cells; the combination of 1 μM ACR plus 5 μM LY294002 significantly suppressed the growth of these cells (Figure 2C). In contrast, the growth of Hc normal hepatocytes was not affected by the combination of these agents; even a combination of high concentrations of ACR (5 μM) plus LY294002 (15 μM) did not inhibit the growth of Hc cells in the present study (Figure 2D).

**Table 1:** Combined effects of ACR and PI3K inhibitors on HLF cells

	**LY294002 concentration**			**BKM120 concentration**		
**ACR concentration**	**(μM)**			**(μM)**		
**(μM)**	**5**	**10**	**15**	**5**	**10**	**15**
**0.5**	**+++**	**+**	**±**	**±**	**++**	**++**
**1**	**+++**	**++**	**±**	**+++**	**++**	**+**
**5**	**-**	**++**	**-**	**-**	**-**	**-**

Note:

“-”, CI1.1-1.3 moderate antagonism;

“±”, CI0.9-1.1 additive effect;

“+”, CI0.8-0.9 slight synergism;

“++”, CI0.6-0.8 moderate synergism;

“+++”, CI0.4-0.6 synergism;

Abbreviations: CI Combination index, ACR Acyclic retinoid.

### ACR plus BKM120 cause synergistic inhibition of growth in HLF cells

**Figure 3:**
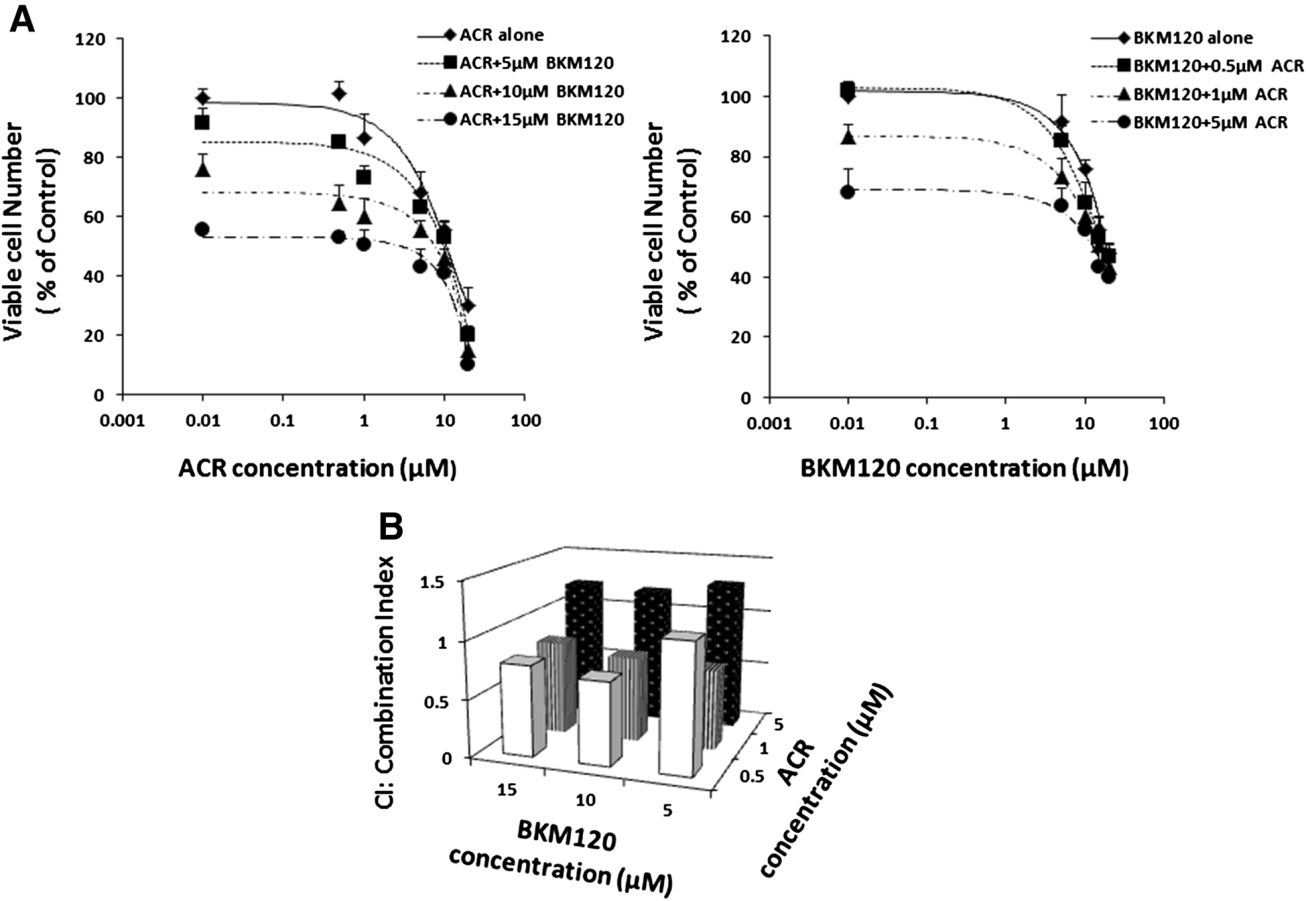
**Inhibition of cell growth by ACR alone, BKM120 alone, or various combinations of these agents in HCC cells. (A)** HLF human HCC cells were treated with the indicated concentrations of ACR alone, BKM120 alone, or various combinations of these agents for 48 hours. Cell viability was determined by the MTS assay and expressed as a percentage of the control value. **(B)** The data obtained in **(A)** was used to calculate the combination index. Error bars present the SD of triplicate assays.

In order to examine whether PI3K inhibitors are promising agents to potently suppress the growth of HCC cells in conjunction with ACR, the combined effects of ACR plus BKM120, another selective PI3K inhibitor [33], on the growth of HLF cells were next investigated. The combination of ACR plus BKM120 significantly inhibited the growth of HLF cells. In particular, when the cells were treated with 1 μM ACR plus 5 μM BKM120, the CI-isobologram analysis yielded a CI-index of 3+ (synergism) (Figure 3A,B, and Table 1). These findings suggest that combination therapy using ACR plus PI3K inhibitors might be an effective regimen for inhibiting the growth of HCC cells.

### ACR plus LY294002 cooperatively induce apoptosis in HLF cells

**Figure 4:**
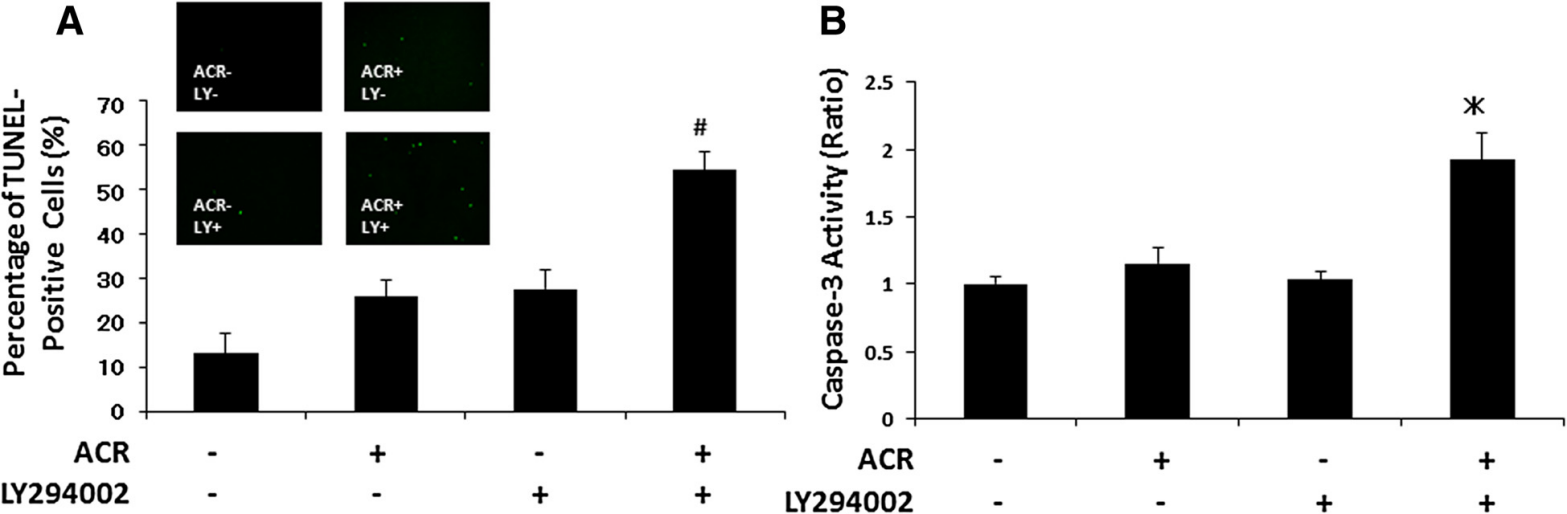
**Effects of the combination of ACR and LY294002 on the induction of apoptosis in HLF cells.** The cells were treated with vehicle, 1 μM ACR alone, 5 μM LY294002 alone, or a combination of 1 μM ACR and 5 μM LY294002 for 48 or 72 hours. **(A)** TUNEL assays were performed using cells treated with test drugs for 48 hours. TUNEL-positive cells were counted and examined as the percentage of the DAPI-positive cell number (500 cells were counted in each flask). **(B)** Caspase-3 activity assays were performed with a fluorometric system using samples treated for 72 hours. # *P* < 0.01. * *P* < 0.05.

The next study examined whether the synergistic growth inhibition in HLF cells induced by treatment with ACR plus LY294002 is associated with the induction of apoptosis. The ratio of TUNEL-positive cells was not significantly increased by treatment with 1 μM ACR alone (26.9%) or 5 μM LY294002 alone (27.6%) in comparison to that of control untreated cells (15.2%). However, when the cells were treated with the combination of these agents, TUNEL-positive cells significantly increased to 54.4% of the total remaining cells (Figure 4A). Similar results were also observed in the caspase-3 activity assay; the combined treatment with ACR plus LY294002 significantly increased the levels of caspase-3 activity in HLF cells, whereas treatment with ACR alone or LY294002 alone did not exert such an effect (Figure 4B).

### ACR plus LY294002 cooperatively suppress the phosphorylation of RXRα, ERK, and Akt and increase the RXRE promoter activity in HLF cells

**Figure 5:**
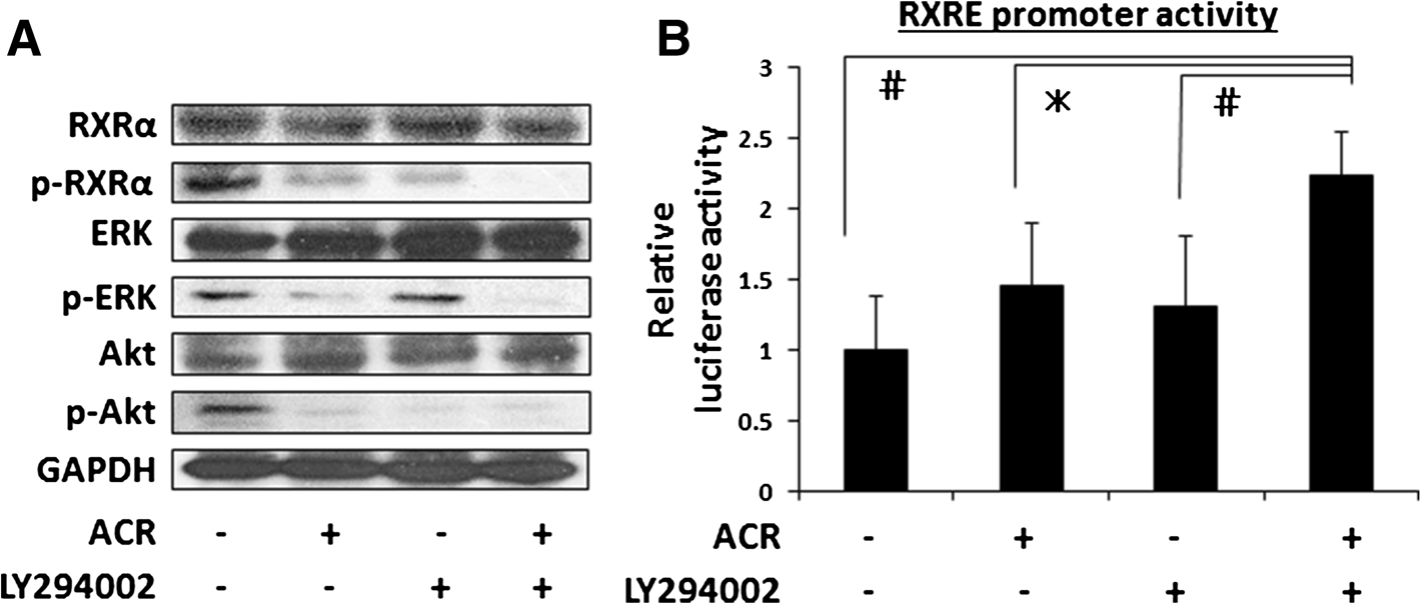
**Effects of the combination of ACR and LY294002 on the phosphorylation of RXRα, ERK, and Akt proteins and the transcriptional activity of the RXRE promoter in HLF cells. (A)** The cells were treated with vehicle, 1 μM ACR alone, 5 μM LY294002 alone, or a combination of 1 μM ACR and 5 μM LY294002 for 12 hours. The extracted proteins were examined by western blot analysis using the respective antibodies. Repeat western blots gave similar results. **(B)** A transient transfection reporter assay was performed with the RXRE luciferase reporter in the presence of vehicle, 1 μM ACR alone, 5 μM LY294002 alone, or a combination of 1 μM ACR and 5 μM LY294002. Relative luciferase activity was determined after 24 hours. Columns and lines indicate the means and SD of triplicate assays. # *P* < 0.01. * *P* < 0.05.

RXRα phosphorylation is involved in the development of HCC, and thus might be a promising target for HCC chemoprevention [4-9]. Therefore, the effects of the combination of ACR and LY294002 on the phosphorylation of RXRα and related signaling molecules were next investigated in HLF cells. As shown in Figure 5A, there was a significant decrease in the expression levels of p-RXRα, p-ERK, and p-Akt proteins when the cells were treated with 1 μM ACR. Treatment with 5 μM LY294002 also caused a marked decrease in the expression levels of p-RXRα and p-Akt proteins in these cells. Moreover, the decrease in the expression levels of p-RXRα, p-ERK, and p-Akt proteins was greater when the cells were treated with a combination of these agents.

In addition, there was a significant increase in the transcriptional activity of the RXRE reporter when HLF cells were treated with a combination of ACR and LY294002, whereas treatment with the same concentrations of ACR alone or LY294002 alone did not upregulate the activity of this promoter (Figure 5B). Because RXRs modulate the expression of target genes by interacting with the RXRE element located in the promoter regions of these genes [1,2], this finding may indicate that LY294002 enhances the transcriptional activity of the RXRE promoter induced by ACR, at least in part by inhibiting the phosphorylation of RXRα.

### ACR and LY294002 cooperatively increase the cellular levels of RARβ and p21^CIP1^, but decrease the levels of cyclin D1, in HLF cells

**Figure 6:**
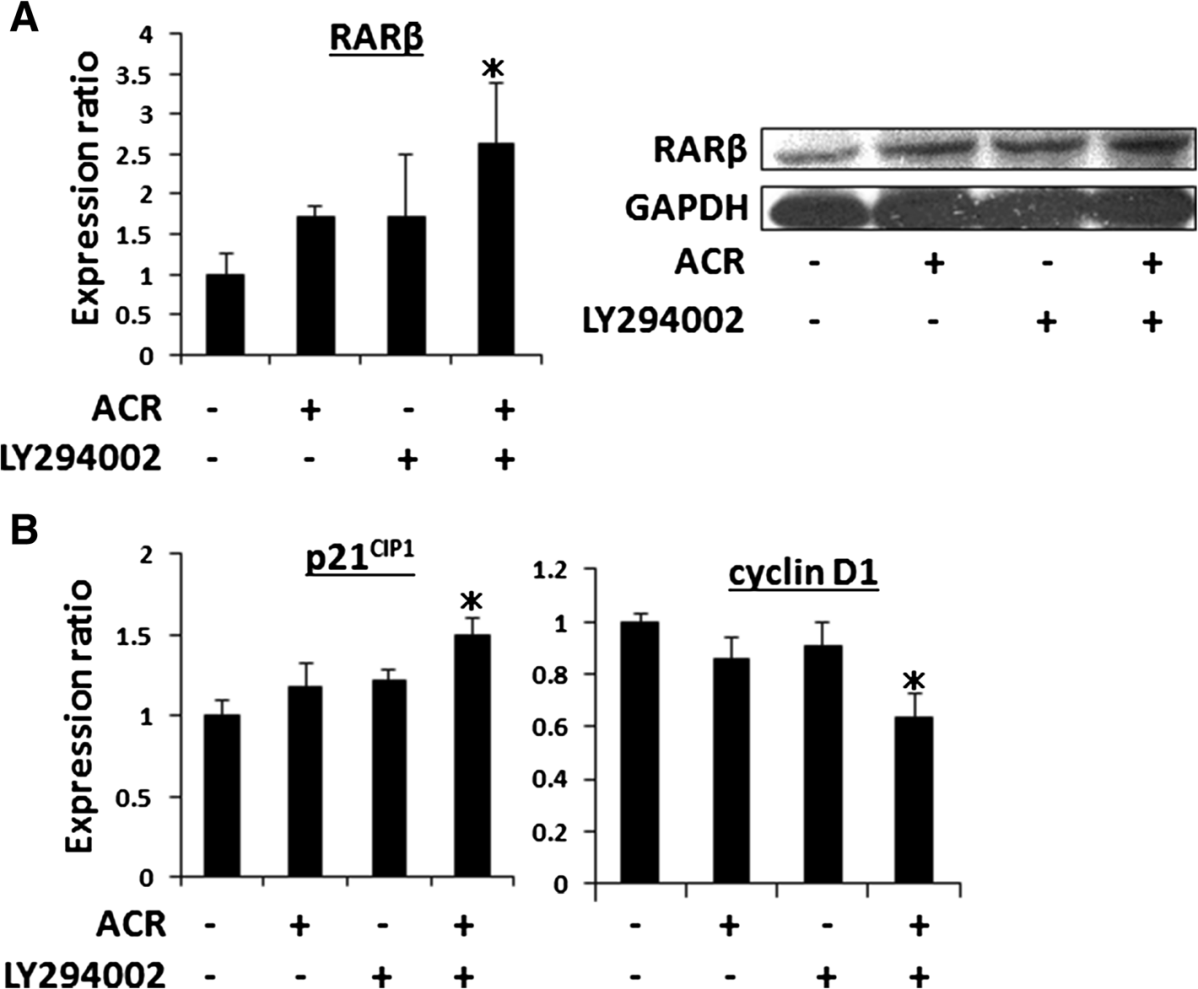
**Effects of the combination of ACR and LY294002 on the cellular expression levels of RARβ, p21**^**CIP1**^**, and cyclin D1 in HLF cells. (A)** The expression levels of RARβ mRNA (left panel) and protein (right panel) were examined by quantitative real-time RT-PCR analysis and western blot analysis, respectively, using cells treated with the test drugs for 24 hours. **(B)** Quantitative real-time RT-PCR analysis to examine the expression levels of p21^CIP1^ and cyclin D1 mRNAs were performed using cells treated with the test drugs for 24 hours. The expression level of each mRNA was normalized to the level of β-actin mRNA. Values represent the means ± SD of triplicate analyses. * *P* < 0.05.

Because the transcriptional activity of the RXRE promoter was significantly increased by treatment with ACR plus LY294002 (Figure 5B), the next study examined whether this combination cooperatively altered the expression of target molecules of ACR, including RARβ, p21^CIP1^, and cyclin D1 [13,25,27,34], in HLF cells. As shown in Figure 6A, the mRNA and protein expression levels of RARβ were significantly increased on combined treatment with ACR and LY294002. Quantitative RT-PCR analyses also revealed that there was a significant increase in the levels of p21^CIP1^ mRNA, but a decrease in the levels of cyclin D1 mRNA, in HLF cells, upon treatment with this combination (Figure 6B).

## Discussion and conclusions

In order to improve the clinical outcome for patients with HCC, development of effective strategies for the chemoprevention and chemotherapy of this malignancy is urgently required. We believe that combination chemoprevention using ACR as a key agent is a promising method for attaining this objective, because it provides an opportunity to take advantage of the synergistic effects of ACR on growth inhibition in HCC cells [17,25-30]. The present study provides the first evidence that the combination of ACR with LY294002, a PI3K inhibitor, synergistically inhibited the growth of human HCC cells through the induction of apoptosis. Activation of the PI3K/Akt pathway, which is common in many cancers such as HCC [21,22], contributes to the inhibition of apoptosis and induction of therapeutic resistance in cancer cells, indicating that targeting this pathway can inhibit the survival and growth of cancer cells through various mechanisms such as potentiation of the effects of chemotherapeutic drugs [18-20,23,24]. For instance, the combination of all-*trans* retinoic acid with LY294002 enhanced growth suppressive effects in leukemic cells by inducing apoptosis [35].

**Figure 7:**
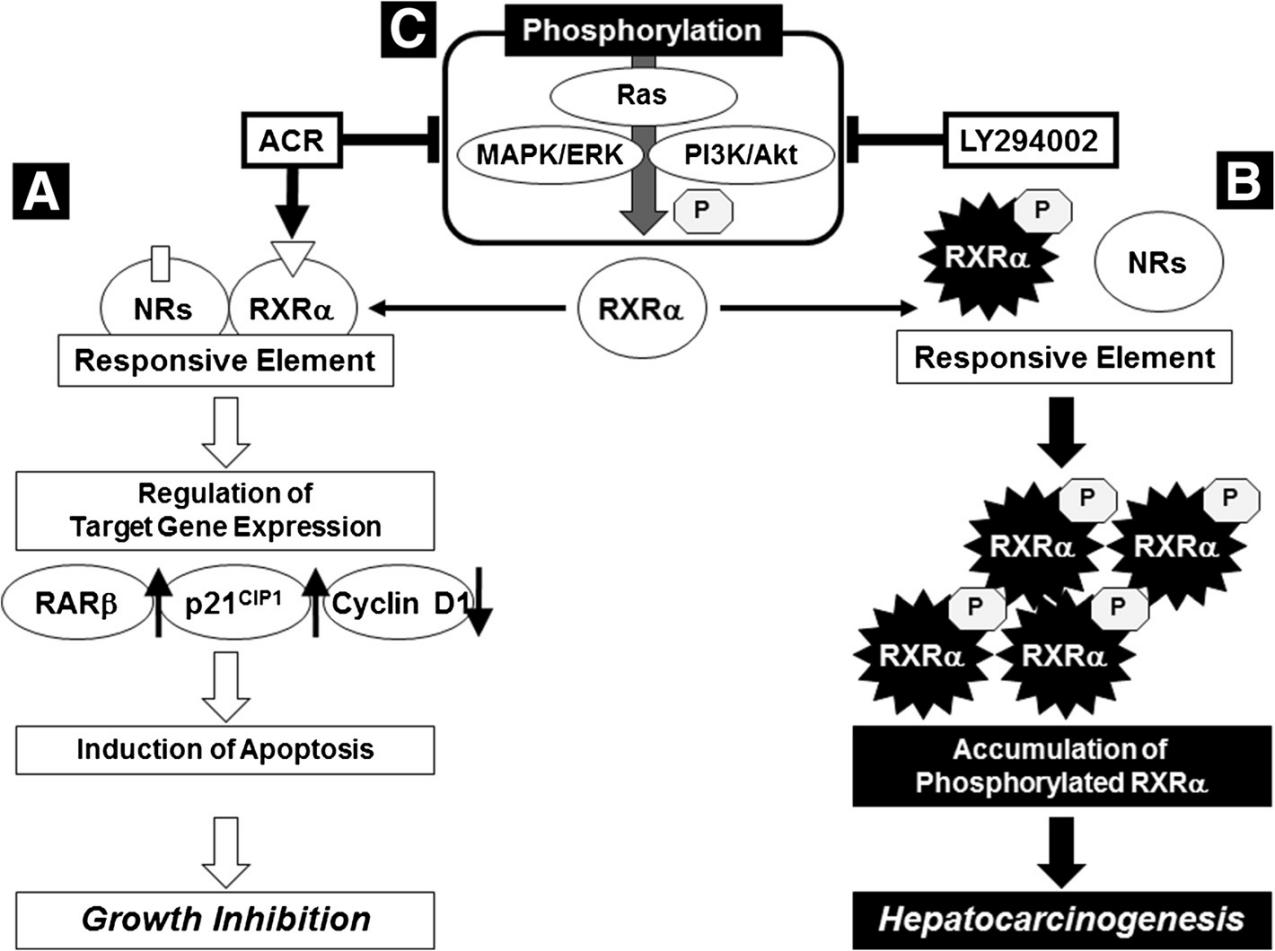
**A hypothetical schematic representation of the effects of the combination of ACR and LY294002 on growth inhibition in HCC cells.** When ACR binds to and activates RXRα, it forms homo- and/or heterodimers with other nuclear receptors (NRs), including RARs. This results in the activation of the transcriptional activity of the responsive element, thus controlling the expression of the target genes, such as RARβ, p21^CIP1^, and cyclin D1, which induce apoptosis and inhibit the growth of HCC cells **(A)**. In HCC cells, the MAPK/ERK and PI3K/Akt pathways, both of which are located downstream of Ras, are highly activated and phosphorylate the RXRα protein. The accumulation of phosphorylated RXRα protein, which impairs dimer formation and the subsequent transactivation functions of this receptor, cause a deviation from normal cell proliferation and differentiation, thereby playing a critical role in liver carcinogenesis **(B)**. ACR and LY294002 inhibit RXRα phosphorylation by inhibiting ERK and Akt phosphorylation, resulting in restoration of receptor function and activation of the transcriptional activity of the responsive element **(C)**. For additional details, see the Discussion section.

The hypotheses that explain the synergism generated by the combination of ACR and LY294002 are summarized in Figure 7. First, it should be noted that phosphorylation of RXRα was markedly inhibited by the combination of ACR and LY294002 in the present study. This finding seems to be significant because RXRα phosphorylation plays a role in the development of HCC and, therefore, might be a critical target for the implementation of HCC chemoprevention [5,7-9]. Accumulation of phosphorylated RXRα induced by the Ras/MAPK activation interferes with the function of normal (unphosphorylated) RXRα in a dominant negative manner [8,9]. This and prior studies [4,17,25,28] show that ACR alone inhibits the phosphorylation of RXRα and ERK proteins in HCC cells. Moreover, in the present study, ACR alone also dephosphorylated the Akt protein in HLF cells. These findings suggest that the combination of ACR and LY294002 cooperatively inhibit the phosphorylation of RXRα through dephosphorylation of ERK and Akt, which leads to the synergistic inhibition of growth and the induction of apoptosis in HCC cells. The results of the present research, together with those of previous studies [17,25,28-30], suggest that dephosphorylation of RXRα might be a key mechanism for ACR-based combination chemoprevention in HCC cells.

Phosphorylated RXRα loses its ability to form heterodimers with RARβ and this is associated with resistance to retinoids [7]. Therefore, restoration of the function of RXRα by inhibiting its phosphorylation is critical to regulate the expression of retinoid target genes [4-9]. In comparison to treatment with ACR alone or LY294002 alone, combined treatment with these agents significantly increased the transcriptional activity of the RXRE reporter in the present study. This combination also significantly altered the expression levels of ACR target genes, such as RARβ, p21^CIP1^, and cyclin D1 mRNA [13,25,27,34]. Particularly, the induction of RARβ by the combination of ACR and LY294002 might play a crucial role in inhibiting the growth of HCC cells because RARβ, which is a receptor for ACR [36], can exert tumor-suppressive effects in cancer cells and thus be considered as a tumor suppressor gene [37].

In this study, the phosphorylation of Akt is inhibited by ACR alone in HLF cells. This finding seems to be of interest because Akt phosphorylation plays a critical role in cell survival, prevention of apoptosis, and progression of cell cycle in various types of tumors, including HCC [21,22]. The precise mechanism by which ACR inhibits the phosphorylation of Akt protein has not been determined. However, we assume that the dephosphorylation of this protein by ACR might be explained by, at least in part, its ability to inhibit growth factor-dependent RTK activity, because Akt is potently phosphorylated by the activation of RTKs [8,9,14,15,18-20]. For instance, ACR inhibits the growth of HCC cells and prevents chemically induced liver tumorigenesis by targeting the transforming growth factor-α/epidermal growth factor receptor (EGFR) axis, which belongs to RTKs [14,15]. Moreover, a recent study showed that retinol inhibited PI3K activity by decreasing the interaction between PI3K and phosphatidylinositol and this was associated with suppression of cell growth in colon cancer cells [38]. These studies suggest that the PI3K/Akt signaling pathway might be a critical target for retinoids to exert their anti-cancer and chemopreventive properties.

In the current study, the combination of ACR and LY294002 significantly inhibited the growth of HLF, Huh7, and Hep3B HCC cells, whereas the growth of HepG2 cells, the other HCC cell line, was not suppressed by this combination. This might be associated with the phosphorylation status of ERK and Akt proteins because the expression levels of p-ERK and p-Akt proteins were increased in HLF, Huh7, and Hep3B cells compared with HepG2 cells [29]. These results, on the other hand, suggest that HCC cells that overexpress p-ERK and p-Akt proteins might be more sensitive targets for combination therapy using ACR and PI3K inhibitors.

Finally, it should be emphasized that combination therapy and prevention are advantageous because, in addition to providing the potential for synergistic effects, they may reduce the opportunity for the development of drug resistance by cancer cells. Several preclinical studies have shown that cancer cells harboring activated Ras mutations appear to be resistant to treatment with PI3K inhibitor alone [23,39]. However, the use of a combination of the PI3K/Akt inhibitor and a MAPK inhibitor significantly exerted anti-cancer effects in *Kars* G12D-driven or EGFR-mutant lung tumors [23,24]. These studies suggest that effective treatment with PI3K inhibitors require concomitant therapies that target RTK/Ras/MAPK signaling and, therefore, ACR, which can inhibit this signaling pathway [8,9,14,15,40], might be a preferable partner for PI3K inhibitors.

In conclusion, the present study indicates that the combination of ACR and LY294002, which can inhibit the phosphorylation of RXRα, causes a synergistic induction of apoptosis and inhibition of cell growth in human HCC cells. The results of our study suggest that this combination might hold promise as a clinical modality for the prevention and treatment of HCC, due to their synergistic effects. In particular, our finding that the combination regimen using 1 μM ACR plus 5 μM LY294002 synergistically inhibits the growth of HCC cells seems to be clinically relevant because this concentration (1 μM) is approximately the same as the plasma concentration of ACR (which ranged from 1 to 5 μM) in a clinical trial that demonstrated the chemopreventive effects of this agent in the recurrence of secondary HCC [10,11].

## Abbreviations

ACR: Acyclic retinoid; CI: Combination index; DMEM: Dulbecco’s modified eagle medium; EGFR: Epidermal growth factor receptor; ERK: Extracellular signal-regulated kinase; GAPDH: Glyceraldehyde 3-phosphate dehydrogenase; HCC: Hepatocellular carcinoma; IFN: Interferon; MAPK: Mitogen-activated protein kinase; PI3K: Phosphatidylinositol 3-kinase; RAR: Retinoic acid receptor; RTK: Receptor tyrosine kinase; RT-PCR: Reverse transcription PCR; RXR: Retinoid X receptor; RXRE: Retinoid X receptor response element; TUNEL: Terminal deoxynucleotidyl transferase-mediated dUTP nick-end labeling.

## Competing interests

The authors declare that they have no competing interests.

## Authors’ contributions

AB, MS, and TO conceived of the study, participated in its design, and drafted the manuscript. AB, MS, TO, YS, MK, and TK performed in vitro experiment. DT performed statistical analysis. HT and HM helped to draft the manuscript. All authors read and approved the final manuscript.

## Pre-publication history

The pre-publication history for this paper can be accessed here:

http://www.biomedcentral.com/1471-2407/13/465/prepub

## References

[B1] Mangelsdorf DJ Thummel C Beato M Herrlich P Schutz G Umesono K Blumberg B Kastner P Mark M Chambon P Evans RM The nuclear receptor superfamily: the second decade Cell 1995 83 835 839 8521507 10.1016/0092-8674(95)90199-x PMC6159888

[B2] Chambon P A decade of molecular biology of retinoic acid receptors FASEB J 1996 10 940 954 8801176

[B3] Altucci L Leibowitz MD Ogilvie KM de Lera AR Gronemeyer H RAR and RXR modulation in cancer and metabolic disease Nat Rev Drug Discov 2007 6 793 810 17906642 10.1038/nrd2397

[B4] Matsushima-Nishiwaki R Okuno M Takano Y Kojima S Friedman SL Moriwaki H Molecular mechanism for growth suppression of human hepatocellular carcinoma cells by acyclic retinoid Carcinogenesis 2003 24 1353 1359 12807734 10.1093/carcin/bgg090

[B5] Matsushima-Nishiwaki R Okuno M Adachi S Sano T Akita K Moriwaki H Friedman SL Kojima S Phosphorylation of retinoid X receptor alpha at serine 260 impairs its metabolism and function in human hepatocellular carcinoma Cancer Res 2001 61 7675 7682 11606411

[B6] Adachi S Okuno M Matsushima-Nishiwaki R Takano Y Kojima S Friedman SL Moriwaki H Okano Y Phosphorylation of retinoid X receptor suppresses its ubiquitination in human hepatocellular carcinoma Hepatology 2002 35 332 340 11826406 10.1053/jhep.2002.31164

[B7] Yoshimura K Muto Y Shimizu M Matsushima-Nishiwaki R Okuno M Takano Y Tsurumi H Kojima S Okano Y Moriwaki H Phosphorylated retinoid X receptor alpha loses its heterodimeric activity with retinoic acid receptor beta Cancer Sci 2007 98 1868 1874 17900311 10.1111/j.1349-7006.2007.00621.x PMC11159768

[B8] Shimizu M Takai K Moriwaki H Strategy and mechanism for the prevention of hepatocellular carcinoma: phosphorylated retinoid X receptor alpha is a critical target for hepatocellular carcinoma chemoprevention Cancer Sci 2009 100 369 374 19068086 10.1111/j.1349-7006.2008.01045.x PMC11159360

[B9] Shimizu M Sakai H Moriwaki H Chemoprevention of hepatocellular carcinoma by acyclic retinoid Front Biosci 2011 16 759 769 10.2741/3718 21196201

[B10] Muto Y Moriwaki H Ninomiya M Adachi S Saito A Takasaki KT Tanaka T Tsurumi K Okuno M Tomita E Nakamura T Kojima T Prevention of second primary tumors by an acyclic retinoid, polyprenoic acid, in patients with hepatocellular carcinoma. Hepatoma prevention study group N Engl J Med 1996 334 1561 1567 8628336 10.1056/NEJM199606133342402

[B11] Muto Y Moriwaki H Saito A Prevention of second primary tumors by an acyclic retinoid in patients with hepatocellular carcinoma N Engl J Med 1999 340 1046 1047 10189289 10.1056/NEJM199904013401315

[B12] Suzui M Masuda M Lim JT Albanese C Pestell RG Weinstein IB Growth inhibition of human hepatoma cells by acyclic retinoid is associated with induction of p21(CIP1) and inhibition of expression of cyclin D1 Cancer Res 2002 62 3997 4006 12124333

[B13] Suzui M Shimizu M Masuda M Lim JT Yoshimi N Weinstein IB Acyclic retinoid activates retinoic acid receptor beta and induces transcriptional activation of p21(CIP1) in HepG2 human hepatoma cells Mol Cancer Ther 2004 3 309 316 15026551

[B14] Nakamura N Shidoji Y Moriwaki H Muto Y Apoptosis in human hepatoma cell line induced by 4,5-didehydro geranylgeranoic acid (acyclic retinoid) via down-regulation of transforming growth factor-alpha Biochem Biophys Res Commun 1996 219 100 104 8619789 10.1006/bbrc.1996.0188

[B15] Kagawa M Sano T Ishibashi N Hashimoto M Okuno M Moriwaki H Suzuki R Kohno H Tanaka T An acyclic retinoid, NIK-333, inhibits N-diethylnitrosamine-induced rat hepatocarcinogenesis through suppression of TGF-alpha expression and cell proliferation Carcinogenesis 2004 25 979 985 14742314 10.1093/carcin/bgh093

[B16] Shimizu M Sakai H Shirakami Y Iwasa J Yasuda Y Kubota M Takai K Tsurumi H Tanaka T Moriwaki H Acyclic retinoid inhibits diethylnitrosamine-induced liver tumorigenesis in obese and diabetic C57BLKS/J- + (db)/+Lepr(db) mice Cancer Prev Res 2011 4 128 136 10.1158/1940-6207.CAPR-10-0163 21071580

[B17] Shimizu M Shirakami Y Sakai H Iwasa J Shiraki M Takai K Naiki T Moriwaki H Combination of acyclic retinoid with branched-chain amino acids inhibits xenograft growth of human hepatoma cells in nude mice Hepatol Res 2012 42 1241 1247 23181540 10.1111/j.1872-034X.2012.01045.x

[B18] Engelman JA Targeting PI3K signalling in cancer: opportunities, challenges and limitations Nat Rev Cancer 2009 9 550 562 19629070 10.1038/nrc2664

[B19] Courtney KD Corcoran RB Engelman JA The PI3K pathway as drug target in human cancer J Clin Oncol 2010 28 1075 1083 20085938 10.1200/JCO.2009.25.3641 PMC2834432

[B20] Vivanco I Sawyers CL The phosphatidylinositol 3-Kinase AKT pathway in human cancer Nat Rev Cancer 2002 2 489 501 12094235 10.1038/nrc839

[B21] Zhou Q Lui VW Yeo W Targeting the PI3K/Akt/mTOR pathway in hepatocellular carcinoma Future Oncol 2011 7 1149 1167 21992728 10.2217/fon.11.95

[B22] Llovet JM Bruix J Molecular targeted therapies in hepatocellular carcinoma Hepatology 2008 48 1312 1327 18821591 10.1002/hep.22506 PMC2597642

[B23] Engelman JA Chen L Tan X Crosby K Guimaraes AR Upadhyay R Maira M McNamara K Perera SA Song Y Chirieac LR Kaur R Lightbown A Simendinger J Li T Padera RF Garcia-Echeverria C Weissleder R Mahmood U Cantley LC Wong KK Effective use of PI3K and MEK inhibitors to treat mutant Kras G12D and PIK3CA H1047R murine lung cancers Nat Med 2008 14 1351 1356 19029981 10.1038/nm.1890 PMC2683415

[B24] Faber AC Li D Song Y Liang MC Yeap BY Bronson RT Lifshits E Chen Z Maira SM Garcia-Echeverria C Wong KK Engelman JA Differential induction of apoptosis in HER2 and EGFR addicted cancers following PI3K inhibition Proc Natl Acad Sci U S A 2009 106 19503 19508 19850869 10.1073/pnas.0905056106 PMC2765921

[B25] Tatebe H Shimizu M Shirakami Y Sakai H Yasuda Y Tsurumi H Moriwaki H Acyclic retinoid synergises with valproic acid to inhibit growth in human hepatocellular carcinoma cells Cancer Lett 2009 285 210 217 19520494 10.1016/j.canlet.2009.05.019

[B26] Obora A Shiratori Y Okuno M Adachi S Takano Y Matsushima-Nishiwaki R Yasuda I Yamada Y Akita K Sano T Shimada J Kojima S Okano Y Friedman SL Moriwaki H Synergistic induction of apoptosis by acyclic retinoid and interferon-beta in human hepatocellular carcinoma cells Hepatology 2002 36 1115 1124 12395321 10.1053/jhep.2002.36369

[B27] Shimizu M Suzui M Deguchi A Lim JT Xiao D Hayes JH Papadopoulos KP Weinstein IB Synergistic effects of acyclic retinoid and OSI-461 on growth inhibition and gene expression in human hepatoma cells Clin Cancer Res 2004 10 6710 6721 15475462 10.1158/1078-0432.CCR-04-0659

[B28] Kanamori T Shimizu M Okuno M Matsushima-Nishiwaki R Tsurumi H Kojima S Moriwaki H Synergistic growth inhibition by acyclic retinoid and vitamin K2 in human hepatocellular carcinoma cells Cancer Sci 2007 98 431 437 17270033 10.1111/j.1349-7006.2006.00384.x PMC11158363

[B29] Tatebe H Shimizu M Shirakami Y Tsurumi H Moriwaki H Synergistic growth inhibition by 9-cis-retinoic acid plus trastuzumab in human hepatocellular carcinoma cells Clin Cancer Res 2008 14 2806 2812 18451248 10.1158/1078-0432.CCR-07-4708

[B30] Ohno T Shirakami Y Shimizu M Kubota M Sakai H Yasuda Y Kochi T Tsurumi H Moriwaki H Synergistic growth inhibition of human hepatocellular carcinoma cells by acyclic retinoid and GW4064, a farnesoid X receptor ligand Cancer Lett 2012 323 215 222 22579649 10.1016/j.canlet.2012.04.015

[B31] Zhao L Wientjes MG Au JL Evaluation of combination chemotherapy: integration of nonlinear regression, curve shift, isobologram, and combination index analyses Clin Cancer Res 2004 10 7994 8004 15585635 10.1158/1078-0432.CCR-04-1087

[B32] Shimizu M Yasuda Y Sakai H Kubota M Terakura D Baba A Ohno T Kochi T Tsurumi H Tanaka T Moriwaki H Pitavastatin suppresses diethylnitrosamine-induced liver preneoplasms in male C57BL/KsJ-db/db obese mice BMC Cancer 2011 11 281 21711565 10.1186/1471-2407-11-281 PMC3146939

[B33] Kirstein MM Boukouris AE Pothiraju D Buitrago-Molina LE Marhenke S Schutt J Orlik J Kühnel F Hegermann J Manns MP Vogel A Activity of the mTOR inhibitor RAD001, the dual mTOR and PI3-kinase inhibitor BEZ235 and the PI3-kinase inhibitor BKM120 in hepatocellular carcinoma Liver Int 2013 33 780 793 23489999 10.1111/liv.12126

[B34] Shimizu M Suzui M Deguchi A Lim JT Weinstein IB Effects of acyclic retinoid on growth, cell cycle control, epidermal growth factor receptor signaling, and gene expression in human squamous cell carcinoma cells Clin Cancer Res 2004 10 1130 1140 14871993 10.1158/1078-0432.ccr-0714-3

[B35] Zhao S Konopleva M Cabreira-Hansen M Xie Z Hu W Milella M Estrov Z Mills GB Andreeff M Inhibition of phosphatidylinositol 3-kinase dephosphorylates BAD and promotes apoptosis in myeloid leukemias Leukemia 2004 18 267 275 14628071 10.1038/sj.leu.2403220

[B36] Yamada Y Shidoji Y Fukutomi Y Ishikawa T Kaneko T Nakagama H Imawari M Moriwaki H Muto Y Positive and negative regulations of albumin gene expression by retinoids in human hepatoma cell lines Mol Carcinog 1994 10 151 158 7519016 10.1002/mc.2940100306

[B37] Alvarez S Germain P Alvarez R Rodriguez-Barrios F Gronemeyer H de Lera AR Structure, function and modulation of retinoic acid receptor beta, a tumor suppressor Int J Biochem Cell Biol 2007 39 1406 1415 17433757 10.1016/j.biocel.2007.02.010

[B38] Park EY Wilder ET Chipuk JE Lane MA Retinol decreases phosphatidylinositol 3-kinase activity in colon cancer cells Mol Carcinog 2008 47 264 274 17918208 10.1002/mc.20381

[B39] Ihle NT Lemos R Jr Wipf P Yacoub A Mitchell C Siwak D Mills GB Dent P Kirkpatrick DL Powis G Mutations in the phosphatidylinositol-3-kinase pathway predict for antitumor activity of the inhibitor PX-866 whereas oncogenic Ras is a dominant predictor for resistance Cancer Res 2009 69 143 150 19117997 10.1158/0008-5472.CAN-07-6656 PMC2613546

[B40] Nakagawa T Shimizu M Shirakami Y Tatebe H Yasuda I Tsurumi H Moriwaki H Synergistic effects of acyclic retinoid and gemcitabine on growth inhibition in pancreatic cancer cells Cancer Lett 2009 273 250 256 18789834 10.1016/j.canlet.2008.08.004

